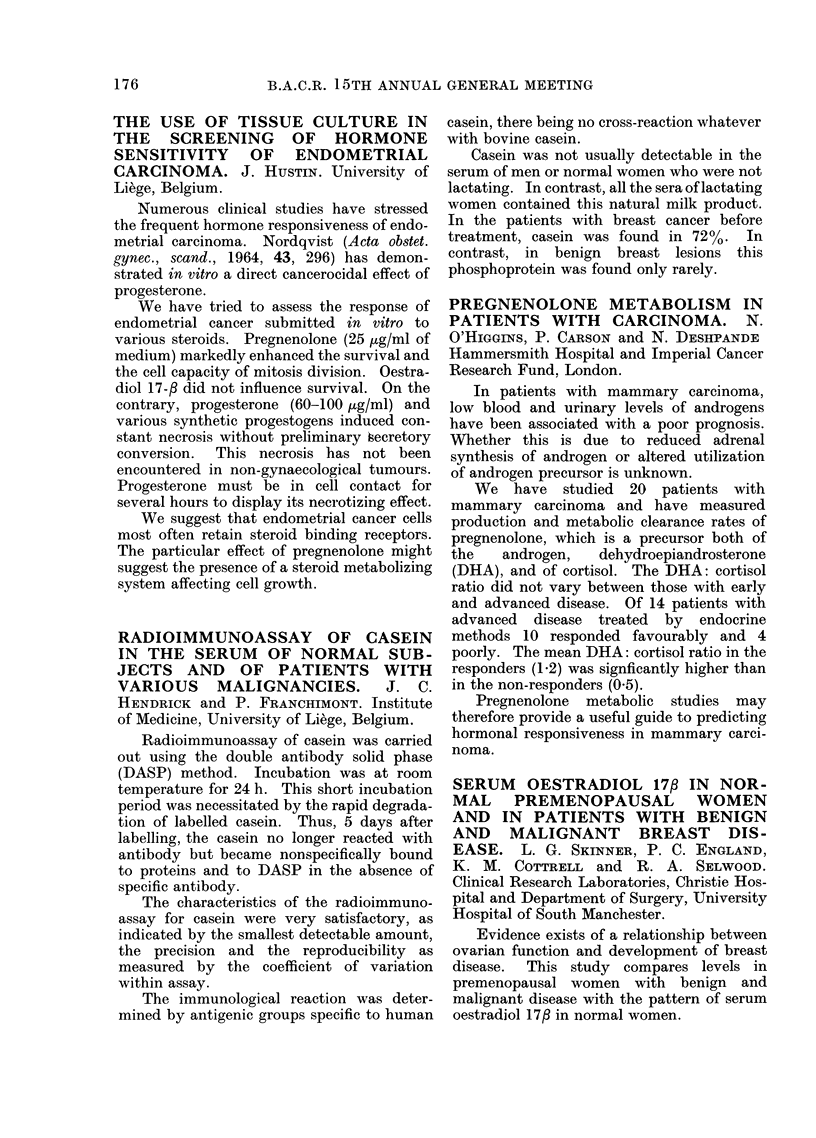# Proceedings: Radioimmunoassay of casein in the serum of normal subjects and of patients with various malignancies.

**DOI:** 10.1038/bjc.1974.142

**Published:** 1974-08

**Authors:** J. C. Hendrick, P. Franchimont


**RADIOIMMUNOASSAY OF CASEIN IN THE SERUM OF NORMAL SUBJECTS AND OF PATIENTS WITH VARIOUS MALIGNANCIES.** J. C. HENDRICK and P. FRANCHIMONT. Institute of Medicine, University of Liège, Belgium.

Radioimmunoassay of casein was carried out using the double antibody solid phase (DASP) method. Incubation was at room temperature for 24 h. This short incubation period was necessitated by the rapid degradation of labelled casein. Thus, 5 days after labelling, the casein no longer reacted with antibody but became nonspecifically bound to proteins and to DASP in the absence of specific antibody.

The characteristics of the radioimmunoassay for casein were very satisfactory, as indicated by the smallest detectable amount, the precision and the reproducibility as measured by the coefficient of variation within assay.

The immunological reaction was determined by antigenic groups specific to human casein, there being no cross-reaction whatever with bovine casein.

Casein was not usually detectable in the serum of men or normal women who were not lactating. In contrast, all the sera of lactating women contained this natural milk product. In the patients with breast cancer before treatment, casein was found in 72%. In contrast, in benign breast lesions this phosphoprotein was found only rarely.